# Understanding fatigue in adults with visual impairment: A path analysis study of sociodemographic, psychological and health-related factors

**DOI:** 10.1371/journal.pone.0224340

**Published:** 2019-10-25

**Authors:** Wouter Schakel, Christina Bode, Peter M. van de Ven, Hilde P. A. van der Aa, Carel T. J. Hulshof, Gerardus H. M. B. van Rens, Ruth M. A. van Nispen

**Affiliations:** 1 Department of Ophthalmology, Amsterdam UMC, Vrije Universiteit Amsterdam, Amsterdam Public Health Research Institute, Amsterdam, The Netherlands; 2 Department of Psychology, Health and Technology, University of Twente, Enschede, The Netherlands; 3 Department of Epidemiology and Biostatistics, Amsterdam UMC, Vrije Universiteit Amsterdam, Amsterdam Public Health Research Institute, Amsterdam, The Netherlands; 4 NetherlandsCoronel Institute of Occupational Health, Amsterdam UMC, University of Amsterdam, Amsterdam Public Health Research Institute, Amsterdam, The Netherlands; 5 Department of Ophthalmology, Elkerliek Hospital, Helmond, The Netherlands; University of Lleida, SPAIN

## Abstract

**Background:**

Fatigue is a disabling problem in patients with visual impairment, but its etiology is still poorly understood. Our objective was to identify the determinants of fatigue in adults with visual impairment compared to adults with normal sight.

**Methods:**

Cross-sectional data on fatigue and sociodemographic, psychological and health-related factors was obtained with validated questionnaires. Structural equational modeling using hypothesized relationships and explorative analyses were used to identify (in)direct pathways contributing to fatigue in 247 adults with visual impairment. The model was then tested in a reference group of 151 adults with normal sight.

**Results:**

The final model explained 64% of fatigue variance in participants with visual impairment and revealed the following factors to be directly associated with fatigue: depressive symptoms (β = 0.723, *p*<0.001), perceived health (β = -0.158, *p =* 0.004), accommodative coping (β = 0.116, *p =* 0.030) and somatic comorbidity (β = 0.311, *p =* 0.001). Self-efficacy demonstrated a beneficial indirect effect on fatigue (β = -0.228, *p*<0.001) mediated by depression, accommodative coping and perceived health. Sleep disorder had an indirect effect on fatigue (β = 0.656, *p*<0.001) mediated by depression and perceived health. After removal of sleep disorder, the model explained 58% of the fatigue variance in normally sighted adults but pathways involving accommodative coping and somatic comorbidity were not confirmed.

**Conclusions:**

These findings suggest that depression and perceived health are important mediating factors that contribute to fatigue in persons with visual impairment and normal sight. In contrast, somatic comorbidity, sleep disorders and accommodative coping seem to have a specific contribution to vision-related fatigue. These factors should be addressed in interventions to assist individuals with visual impairment in dealing with fatigue.

## Introduction

Fatigue is a major problem in patients with visual impairment that seems to be related to vision loss [1, 2]. In previous work, it was shown that adults with visual impairment experience greater fatigue severity and were four times more likely to endure severe impact of fatigue on daily life compared to a population with normal sight. These symptoms were associated with increased societal costs and explained a substantial proportion of the economic burden of low vision through loss in work participation [3]. Screening and successful treatment of fatigue in low vision rehabilitation is therefore of paramount importance for the wellbeing of the patient and for society as a whole. However, the etiology of fatigue related to visual impairment has not yet been investigated, and evidence-based treatment options are lacking.

Studies on patient populations with other chronic diseases have described various factors that contribute to the multidimensional nature of fatigue in great detail. Fatigue can be a primary symptom of the disease itself caused by underlying disease-specific mechanisms, such as pain in rheumatoid arthritis [4] and inflammatory processes in multiple sclerosis [5]. With the exception of uveitis or some other acute ophthalmic problems, ocular pain is often not an issue (anymore) in persons with irreversible vision loss and is therefore expected to be less relevant for vision-related fatigue. Evidence from multiple reviews highlight the importance of psychosocial and behavioral factors that can maintain fatigue and related disability in chronic disorders [6–8]. A recent study identified demographic variables, motivational and concentration problems, pain, sleep disturbances, physical functioning, reduced activity and lower self-efficacy as the most important factors explaining fatigue among a large dataset of patients with various common chronic disorders [9]. These psychosocial factors and health-related symptoms were generic for the various chronic diseases included and may therefore also be important for fatigue in patients with visual impairment.

Fatigue in relation to chronic disease is often acknowledged to be determined by a complex interplay between various factors. Several cross-sectional studies have investigated how these interrelationships contribute to fatigue using structural equational modeling (SEM) techniques. For example, the path model of Nicassio et al. identified an indirect association between disease activity and fatigue, which was mediated by mood disturbance in patients with rheumatoid arthritis [10]. Furthermore, self-efficacy has been demonstrated to assert an indirect effect on fatigue with depressive symptomatology as a mediator in path models for breast cancer survivors and patients with multiple sclerosis [11, 12]. Whether perpetuating factors assert a direct influence or operate through interlinked relationships with one another remains unclear for vision-related fatigue. Therefore, a modeling approach seems most warranted for exploration of this apparent multidimensional concept.

Having a visual impairment negatively affects many aspects of patients’ lives such as societal participation [13] and the ability to perform daily activities [14]. In comparison with individuals with normal sight, patients with visual impairment report reduced quality of life [15] and increased mental health problems such as depression and anxiety [16]. As previous studies have emphasized the importance of psychosocial factors with regard to fatigue in chronic disease, we hypothesize that these associations will be more pronounced in the target population compared to individuals with normal sight. Assessment of the determinants of fatigue in patients with visual impairment may be of benefit for future prospects for treatment and disability management. Since not much is known about the etiology of vision-related fatigue, this study aimed to explore and develop a multidimensional path model examining potential determinants of fatigue severity and impact on daily life in adults with visual impairment. By testing this model in a sample of adults with normal sight, we also aimed to identify factors that generally contribute to fatigue and factors that are specific to people with visual impairment.

## Methods

### Design

In this cross-sectional study, data of adults with visual impairment and adults with normal sight were collected by two researchers through structured telephone interviews and online surveys, respectively. The study was approved by the Medical Ethics Committee of Amsterdam University Medical Centers, Vrije Universiteit Amsterdam, the Netherlands and has been performed according to the principles of the Declaration of Helsinki. All participants gave written informed consent prior to participation.

### Sample and recruitment target population

Patients with visual impairment registered at two multidisciplinary low vision rehabilitation centers in the Netherlands, i.e. Royal Dutch Visio and Bartiméus, were invited to participate between August 2015 and June 2016. Eligibility criteria for these centers are described in the Dutch guideline ‘Vision disorders, rehabilitation and referral’, defining visual impairment as best-corrected decimal visual acuity in the better eye of ≤0.30, and/or visual field of ≤0.30 around the central fixation point, or other severe visual field defects (WHO criteria) [17]. Invitations and consent forms were sent by letter with large print and patients were subsequently informed by telephone if they showed written interest in the study. Patients were considered eligible if they had visual impairment according to the WHO criteria [18], were ≥18 years, had sufficient mastery of the Dutch language, and were not cognitively impaired as assessed by a 6-item version of the Mini Mental State Examination (MMSE) [19]. To ensure that experienced fatigue was related to vision loss rather than other illnesses, we excluded participants diagnosed or treated in the last year for the following chronic conditions known for fatigue symptomatology: 1) cancer, 2) multiple sclerosis, 3) chronic fatigue syndrome, and 4) psychiatric disorders (screened with a single question: “Are you currently being treated for …, or did you receive treatment for … in the past year?”). Out of 1271 invited patients, 321 agreed to participate and gave their written informed consent (25% participation rate). Data of 247 participants were included in the analyses: 59 did not meet the eligibility criteria (3 insufficient mastery of the Dutch language, 56 had chronic physical and/or psychiatric diseases),10 could not be contacted after multiple attempts and 5 refrained from participation. The most common reasons reported for declining participation of contacted non-responders were (in order of highest relevance): too burdensome/intensive, not interested, already participating in another study and unknown reasons.

### Sample and recruitment reference group

Adults with normal sight were recruited through the researcher’s social networks and through advertisements on social media of our clinic. A snowball sampling technique was then used to identify additional study participants. Persons who showed interest were asked to distribute a study information flyer to potential participants among their family and friends. Eligibility criteria were similar to the target population with regard to age, mastery of the Dutch language, cognitive ability and comorbid conditions associated with fatigue. To ensure normal vision, participants were excluded if they had insufficient self-reported vision ability as assessed by a subset of vision questions originating from the Organization for Economic Cooperation and Development long-term disability indicator [20]. In addition, participants were excluded if they were diagnosed or received treatment for a variety of causes of visual impairment: macular degeneration, cataract, glaucoma, diabetic retinopathy, retinal vein occlusion, hemianopia or other eye diseases. A total of 253 adults with normal sight showed interest in the study, of which 233 gave written informed consent and completed the online survey (2 refrained, for 18 almost all data were missing). Preparatory analyses revealed a bimodal distribution of age in the reference group due to a large subset of relatively young versus relatively old participants (mean age ‘younger’ participants: 26.4 ± 4.2 years, range 18–35, *n* = 92; mean age ‘older’ participants: 57.1 ± 8.8 years, range 36–78, *n* = 141). In order to achieve matching samples in terms of their age, five times 10 out of the 92 younger participants were randomly selected, resulting in five data sets of 151 adults with normal sight.

### Preparing potential mediators and outcome measures

Fatigue was defined as a latent variable with two indicators: severity as measured by the Fatigue Assessment Scale (FAS) [21] and impact on daily life as measured by the Modified Fatigue Impact Scale (MFIS) [22]. The following (mental) health-related factors were expected to mediate fatigue: depressive symptoms, self-efficacy, participation (behavioral measure: hours spent on vocational activities and frequency of leisure and social activities), accommodative coping (i.e. flexibly adjusting personal goals to situational constraints and challenges), assimilative coping (i.e. striving to maintain goals by altering unsatisfactory life circumstances in accordance with personal preferences), goal re-engagement coping (i.e. pursuing new goals when personal goals become unattainable), goal dis-engagement coping (i.e. disengaging from personal goals when they become unattainable), perceived health status and sleep disorders (i.e. presence of insomnia, hypersomnia or circadian rhythm sleep disorder). An overview of questionnaires used including measurement properties are presented in Table 1.

**Table 1:** Characteristics of the finalized outcome measures adjusted by IRT analyses used in this study.

Measure		Characteristics			Fit indices					Adjustments		
		Items	Score range	Likert-scale responses	M2	RMSEA	SRMR	TLI	CFI	Theta range	No. items deleted	Collapsed categories
Fatigue Assessment Scale	FAS	10	10–50	5	0.194	0.033	0.078	0.973	0.991	-1.23, 2.66	2	4 + 5
Modified Fatigue Impact Scale	MFIS	21	0–84	5	<0.001	0.068	0.073	0.964	0.970	-2.09, 2.95	3	4 + 5
Patient Health Questionnaire	PHQ-9	9	0–27	4	<0.001	0.070	0.072	0.935	0.961	-1.23, 2.66	1	3 + 4
General Self Efficacy Scale ^a^	G-SES-12	12	12–60	5	<0.001	0.086	0.110	0.556	0.733			
Tenacious Goal Pursuit	TGP	15	15–75	5	<0.001	0.040	0.087	0.936	0.950	-2.36, 2.91	1	1 + 2
Flexible Goal Adjustment	FGA	15	15–75	5	<0.001	0.068	0.068	0.840	0.893	-2.66, 2.41	4	1 + 2
Utrecht Scale for Evaluation- of Rehabilitation-Participation ^a^	USER-P	12	0–100	6	<0.001	0.169	0.102	1.867	0.761			
Goal adjustment Scale: reengagement ^a^ ^b^	GAS-Re	6	6–30	5								
Goal adjustment Scale: disengagement ^a^ ^b^	GAS-De	4	4–20	5								
EuroQol 5 Dimensions 5 Levels ^a^ ^b^	EQ-5D-5L	5	0–1	5								
Holland Sleep Disorder Questionnaire	HSDQ											
Insomnia ^c^	HSDQ-I	8	1–5	5								
Hypersomnia ^d^	HSDQ-H	6	1–5	5								
Circadian rhythm sleep disorder ^e^	HSDQ-C	6	1–5	5								

RSMEA root mean square error approximation, SRMR standardized root mean residual, CFI comparative fit index, TLI Tucker-Lewis index

^a^ included as summary scores in SEM analyses

^b^ insufficient amount of items necessary for IRT analyses using GRM

^c^ insomnia diagnosis: HSDQ-I score >3.68

^d^ hypersomnia diagnosis: HSDQ-H score >2.90

^e^ circadian rhythm sleep disorder diagnosis: HSDQ-C score >3.41.

The majority of the outcome measures were analyzed with item response theory (IRT) models to ensure they had satisfactory psychometric properties. Holland Sleep Disorder Questionnaire (HSDQ) cut-off values were used to measure the presence of sleep disorders and this questionnaire was therefore excluded from IRT analysis [23]. First, individual item analysis was performed to evaluate missing responses (deletion of items with >40% missing values), floor and ceiling effects (deletion of items with >70% scores in highest or lowest response categories), and inter-item correlation (inter-item correlation >0.7 were considered problematic). Next, assumptions of unidimensionality, local independence and monotonicity were checked prior to IRT analysis. Analyses were performed with R using the ltm and mirt packages [24, 25]. A graded response model (GRM) was fitted, which is most commonly used in IRT analyses. Overall fit of the GRM was assessed with the root mean square error approximation (RMSEA [26]; values ≤0.06 represent good fit [27]), the standardized root mean residual (SRMR; values ≤0.08 represent good fit [27]), the comparative fit index (CFI [28]; values ≥0.95 represent good fit [27]) and the Tucker-Lewis index (TLI; values ≥0.95 represent good fit [27]). Questionnaires were adjusted by deleting items and/or collapsing response categories based on the results of item analysis, evaluation of assumptions and GRM analysis. After improving the questionnaires, respondents’ thetas were calculated, representing an interval score of the underlying latent trait. For example, thetas for fatigue severity ranged from -1.23 to 2.66, with scores close to -1.23 representing less severe fatigue and scores closer to 2.66 indicating more severe fatigue. Questionnaire adjustments, GRM model fit and calculated theta ranges are summarized in Table 1. For the Goal Adjustment Scale (GAS) re-engagement and disengagement subscales it was not possible to perform IRT analyses using GRM due to the limited number of items. Also for the General Self Efficacy Scale (GSES-12) and Utrecht Scale for Evaluation of Rehabilitation-Participation (USER-P) it was not possible to fit an unidimensional model because of problematic monotonicity and violation of unidimensionality, respectively. Consequently, for these abovementioned questionnaires summary scores were used as they provide the most suitable estimates when interval scaling is not possible.

### Measurement of independent variables

Socio-demographic characteristics were collected by self-reported questions about age, gender, living situation (living alone / living together with a partner or family), education and employment status. Somatic comorbidity was measured with questions for seven large condition groups: asthma or chronic obstructive pulmonary disease; osteoarthritis and rheumatoid arthritis; peripheral arterial disease; diabetes mellitus; cardiac disease; cerebrovascular accident or stroke; cancer; and other chronic somatic or psychiatric conditions. Because of skewed data, responses were used to define comorbidity by two levels: having no comorbidity or being treated for one or more comorbid chronic conditions. Educational level was expressed as years spent in the educational system.

### Statistical analyses

Descriptive statistics were performed using SPSS version 22.0.0.0. Demographic differences between participants with visual impairment and those with normal sight were evaluated with independent samples t-tests for continuous variables, chi square tests for categorical variables, and non-parametric tests in case of non-normally distributed data. Data and analyses for the five generated subsets of the reference group were pooled by using Rubin’s rules [29]. Univariate associations between all variables were analyzed with Pearson and Spearman’s rho correlations for continuous variables and categorical variables, respectively.

The assumptions of normality and collinearity were checked prior to SEM analysis and found appropriate. Separate path models were developed within a SEM framework to explore direct and indirect pathways for fatigue defined as a latent variable with two indicators (i.e. MFIS and FAS). Analyses were performed with Mplus version 7.4 [30]. Direct and indirect effects were calculated using maximum likelihood estimation based on the delta method, which is robust to non-normality (MLR) and appropriate for continuous and dichotomous variables [31]. Mediation was investigated by inspection of the estimated direct, indirect and total effects within the path models. A distinction was made between (independent) exogenous variables that were not affected by other factors in the model, and endogenous (potential mediating) variables of which variability was expected to be explained by the exogenous variables. First, all variables were entered in an exploratory model for the sample with visual impairment containing both direct and indirect effects. Next, the size of the model was reduced by removing variables that were not significantly related to fatigue in a step-wise procedure prioritizing pathways with smallest p-values and betas. Each new model was re-investigated using multiple fit criteria: χ^2^-goodness-of-fit, RMSEA [26], SRMR, CFI [28] and TLI. RSMEA values close to 0.06 or lower and SRMR values around 0.08 or lower were indicative of good model fit, CFI and TLI values should be close to 0.95 or higher. Additional adjustments were considered by inspection of the modification indices to improve model fit. After specification of the final exploratory model, the model was expanded to include additional theoretically meaningful relationships between psychosocial variables and fatigue resulting in the final model. To this end the correlation matrix was further explored to identify variables with strong associations with both fatigue and identified determinants from the exploratory model. Based on that, sleep and self-efficacy were selected for further analyses and were hypothesized to play a role in fatigue by their interrelationships with depression, accommodative coping and perceived health. Finally, the hypothesized adjusted model for vision-related fatigue was tested in the reference group subsets. In case of acceptable fit, model results of both samples were compared with regard to direction and significance of the included pathways (significant at p<0.05) and their corresponding effect sizes (comparison of standardized beta coefficients).

## Results

### Participants

Socio-demographic characteristics of both samples are shown in Table 2. There were significantly more persons with paid employment, more persons without somatic comorbidity, less persons living together and less persons with sleep disorders in the reference group. Furthermore, mean education in years was significantly higher compared to the sample with visual impairment. As expected, there were significant group differences for the majority of the potential mediators with regard to fatigue severity, fatigue impact, depressive symptoms, frequency of participation, goal re-engagement coping, accommodative coping and perceived health status. On average, participants with visual impairment experienced greater severity and impact of fatigue, reported more symptoms of depression, and had greater tendencies of self-efficacy, goal re-engagement coping and accommodative coping. In contrast, frequency of participation and perceived health status was significantly higher for participants with normal sight. Correlation coefficients between dependent variables and potential mediating variables ranged from -0.05 to 0.66 for patients with visual impairment, and from -0.01 to 0.58 for adults with normal sight (see Table 3).

**Table 2:** Demographics and characteristics of all included variables for participants with visual impairment (n = 247) and normal sight (n = 151).

Construct	Variables	Visual impairment (*n* = 247)		Normal sight (*n* = 151)		*P*
		Mean	SD	Mean	SD	
**Demographics**	Age	57.10	14.30	55.10	11.50	0.132
	Female gender (*n*, %)	152.00	62%0	107.00	71%0	0.055
	Education in years	11.80	02.90	13.70	02.40	<0.001
	Living together (*n*, %)	152.00	62%0	110.00	73%0	0.018
	Somatic comorbidity ≥1 (*n*, %)	118.00	48%0	41.00	27%0	<0.001
	Paid employment (*n*, %)	58.00	23%0	119.00	79%0	<0.001
**Fatigue**	Severity (FAS) sum score	23.20	06.40	18.00	04.40	<0.001
	Severity (FAS) theta	0.35	00.87	-0.38	00.72	<0.001
	Impact (MFIS) sum score	31.40	16.90	19.60	13.20	<0.001
	Impact (MFIS) theta	0.48	01.05	-0.20	00.85	<0.001
**Depressive symptoms**	(PHQ-9) summary score	6.12	03.75	2.23	02.32	<0.001
	(PHQ-9) theta	0.47	00.79	-0.47	00.68	<0.001
**Self-efficacy**	(GSES) sum score	45.20	07.64	46.80	05.75	0.025
**Participation**	Frequency (USER-P) summary score	38.40	08.20	40.50	07.70	0.013
**Goal-adjustment strategies**	Goal re-engagement (GAS) sum score	23.70	04.20	22.30	03.20	<0.001
	Goal disengagement (GAS) sum score	11.10	03.20	11.30	02.50	0.632
	Accommodative coping (FGA) sum score	54.10	08.40	52.60	06.90	0.069
	Accommodative coping (FGA) theta	0.26	00.99	-0.26	00.75	<0.001
	Assimilative coping (TGP) sum score	49.40	08.40	49.30	07.30	0.892
	Assimilative coping (TGP) theta	0.01	00.97	-0.09	00.80	0.276
**Perceived health status**	(EQ-5D-5L) index score	0.80	00.16	0.90	00.11	<0.001
**Sleep**	Sleep disorder (HSDQ) ^a^ (*n*, %)	30.00	12%0	2.00	01%0	<0.001

Estimates and statistics for the reference group were pooled over the five subsets

SD standard deviation, FAS Fatigue Assessment Scale, MFIS Modified Fatigue Impact Scale, PHQ-9 Patient Health Questionnaire, GSES General Self-Efficacy Scale, USER-P Utrecht Scale for Evaluation of Rehabilitation-Participation, GAS Goal Adjustment Scale, FGA Flexible Goal Adjustment scale, TGP Tenacious Goal Pursuit scale, EQ-5D-5L EuroQol 5 Dimensions 5 Levels, HSDQ Holland Sleep Disorder Questionnaire.

^a^ insomnia (HSDQ-I score >3.68) and/or hypersomnia (HSDQ-H score >2.90) and/or circadian rhythm sleep disorder (HSDQ-C score >3.41).

**Table 3:** Intra-correlations for dependent variables (Y), potential mediating variables (X) and independent variables (E) used in the study.

		Y1	Y2	X1	X2	X3	X4	X5	X6	X7	X8	X9	E1	E2	E3	E4	E5	E6
Y1	Fatigue severity ^a^		.72**	.64**	-.35**	-.18**	-.19**	-.09	-.17**	-.17**	-.36**	.23**	.08	-.08	-.10	-.04	.10	.04
Y2	Fatigue impact ^a^	.69**		.66**	-.21**	-.08	-.05	-.08	-.05	-.07	-.43**	.25**	.14*	.02	-.07	-.03	.20**	.01
X1	Depressive symptoms ^a^	.58**	.57**		-.38**	-.09	-.17*	-.23**	-.29**	-.18**	-.41**	.27**	.09	-.09	-.04	-.01	-.04	-.08
X2	Self-efficacy	-.39**	-.40**	-.46**		.26**	.31**	.06	.53**	.63**	.24**	-.07	.04	-.04	.17**	-.05	.05	-.06
X3	Frequency of participation	-.04	-.03	-.07	.33**		.14*	-.09	.07	.17**	.14*	-.10	.02	-.26**	.32**	-.02	-.17**	.32**
X4	Goal re-engagement	-.14**	-.11**	-.05	.23**	.20*		.18**	.46**	.19**	.10	.09	.09	-.10	.06	.01	.02	-.01
X5	Goal dis-engagement	-.01	.08	-.04	-.11	-.01	.32**		.25**	-.13*	.12	.02	-.03	.08	-.11	.01	-.03	.07
X6	Accommodative coping ^a^	-.14**	-.23**	-.14	.48**	.24**	.22**	-.09		.50**	.17**	.04	.16*	.05	-.13*	-.07	.12	.10
X7	Assimilative coping ^a^	-.23**	-.29**	-.18*	.61**	.16*	-.04	-.34**	.42**		.09	.01	-.02	-.09	-.01	-.12	.05	-.07
X8	Perceived health status	-.53**	-.51**	-.52**	.38**	.13	.09	.09	.21*	.16		-.21**	-.13*	-.07	.17**	.03	-.21**	-.06
X9	Sleep disorder	.17*	17.*	.15	.08	-.02	.08	-.13	.17*	.14	-.11		.12	.03	-.28**	-.01	.07	.06
E1	Gender	.09	.11	.19*	-.10	.09	.18*	-.03	.08	-.03	-.16	-.05		-.04	-.14*	-.10	.12	-.01
E2	Age	-.16*	-.06	-.31**	.07	-.07	.03	.08	-.01	-.05	-.02	-.12	-.15		-.19**	.10	.23**	-.37**
E3	Education in years	-.07	-.07	-.02	.10	.33**	.17*	.06	.04	-.06	.04	-.15	-.11	-.02		.10	-.16*	.29**
E4	Living situation	.02	.07	.07	-.14	-.09	-.05	.09	-.16*	-.08	-.12	.07	-.19*	-.13	.01		-.06	-.05
E5	Somatic comorbidity	.19*	.29**	.20*	-.06	-.04	-.11	.05	-.14	-.07	-.29**	-.07	.07	.12	.04	-.07		-.22**
E6	Working status	-.15	.01	-.06	.02	-.15	.02	.07	-.07	-.10	-.12	-.06	.02	-.50**	-.02	-.20*	-.22**	

Upper diagonal: sample with visual impairment (*n* = 247), lower diagonal: best fitting subset of adults with normal sight (*n* = 151), Pearson correlations for pairs of continuous variables, Spearman’s rho correlations in case of at least one categorical variables

* *p* < 0.05

** *p* < 0.01

^a^ outcomes expressed in thetas.

### Exploratory path model for participants with visual impairment

**Fig 1:**
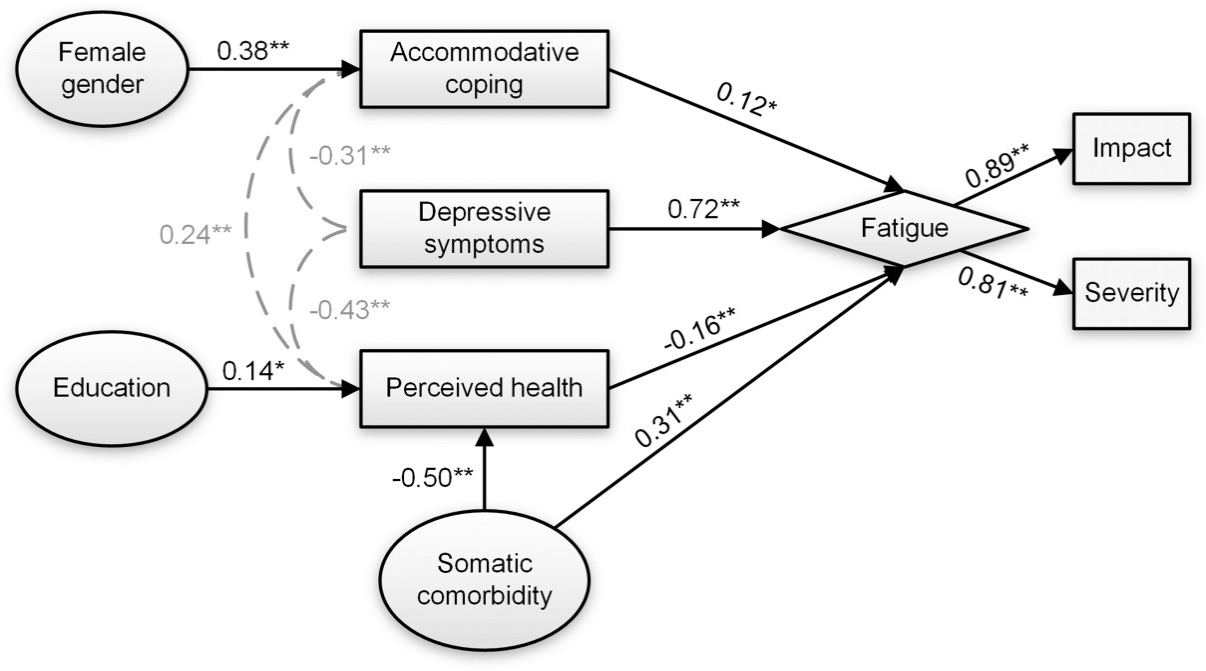
Path analysis output for the final exploratory model in the sample with visual impairment (*n* = 247). Direct effects are represented by arrows with standardized regression coefficients (StdYX for continuous variables, StdY for dichotomous variables. Grey dashed lines signify standardized residual covariance between variables. Ellipses represent independent variables. Rectangles represent psychosocial constructs. Constructs of the latent fatigue variable (diamond shape) are shown in dotted boxes. * *p*<0.05. ** *p*<0.01.

Direct pathways from all endogenous and exogenous variables to fatigue, and indirect pathways from all exogenous variables via the endogenous variables, were initially included in the path analysis. The exploratory model resulted in poor data fit which was expected considering the amount of included variables and number of examined pathways (Table 4). Inspection of the results (S1 Table) revealed significant direct associations between the latent fatigue variable and somatic comorbidity, depression, accommodative coping and perceived health. In addition, accommodative coping mediated the effect between gender on fatigue, education and comorbidity were indirectly associated with fatigue and mediated by perceived health. Next, due to insignificant effects between fatigue and age, gender, education, living status, employment, self-efficacy, participation, goal re-engagement coping, assimilative coping and sleep disorder, these variables were removed. All direct and indirect pathways to fatigue remained significant in the re-specified model, but model fit was still inadequate (Table 4: reduced exploratory model). Further model refinements were made by adding residual covariance between depression and accommodative coping, between depression and perceived health, and between perceived health and accommodative coping, resulting in adequate fit (Table 4: final exploratory model). The standardized path coefficients for the final exploratory model are shown in Fig 1, accounting for 64% of the total variance of fatigue. Table 5 shows that in the final exploratory model more depressive symptoms, greater tendencies of accommodative coping, lower perceived health and the presence of somatic comorbidity were all directly related to greater fatigue.

**Table 4:** Model progression.

Model		χ^2^		SRMR	CFI	TLI	RSMEA		
	χ^2^	*p* value	*df*				estimate	90% CI	p*
**First exploratory model**	504	<0.001	50	0.128	0.489	-0.176	0.192	0.177, 0.207	<0.001
In the first exploratory model, all 6 independent variables (age, gender, education, living situation, comorbidity, employment) and 7 potential mediators (depressive symptoms, self-efficacy, participation, goal re-engagement, goal disengagement, accommodative coping, assimilative coping) were included in the model to explore their (in)direct relationships with the latent fatigue variable									
**Reduced exploratory model**	93	<0.001	15	0.111	0.821	0.702	0.145	0.118, 0.174	<0.001
The reduced exploratory model was derived from the first exploratory model using a stepwise removal of age, gender, living situation, employment self-efficacy, participation, goal re-engagement, goal dis-engagement, assimilative coping and sleep disorder due to insignificant associations with fatigue									
**Final exploratory model**	21	0.065	13	0.042	0.981	0.963	0.051	0.000, 0.089	0.434
The final exploratory model was obtained from the reduced exploratory model by adding correlations for the residual covariance between depression and perceived health, between depression and accommodative coping, and between perceived health and accommodative coping.									
**Final model**	30	0.012	15	0.041	0.973	0.945	0.064	0.029, 0.097	0.223
The final model was derived from the final exploratory model by adding hypothesized pathways of self-efficacy on depression, accommodative coping and perceived health, assumed pathways of sleep on depression and perceived health, and a direct pathway from accommodative coping to depression. Education was removed, residual covariance between depression and perceived health was allowed to correlate.									
**Model examination reference group** ^a^									
Worst fitting subset estimate	46	<0.001	16	0.073	0.915	0.841	0.112	0.075, 0.150	0.004
Best fitting subset estimate	39	<0.001	16	0.066	0.934	0.875	0.098	0.059, 0.137	0.024
**Model examination excluding sleep disorder** ^a^									
Worst fitting subset estimate	22	0.033	12	0.062	0.966	0.928	0.076	0.021, 0.124	0.172
Best fitting subset estimate	18	0.108	12	0.053	0.979	0.956	0.059	0.000, 0.110	0.350

χ^2^ Chi-square test of model fit, *df* degrees of freedom, SRMR standardised root mean residual, CFI comparative fit index, TLI Tucker-Lewis index, RSMEA root mean square error approximation, CI confidence interval

* probability that RMSEA ≤0.05

^a^ results presented as fit range of the worst and best values ​out of 5 separate subsets.

**Table 5:** Standardized direct and indirect path coefficients of the final exploratory model for adults with visual impairment (*n* = 247).

**Direct effects**	β	SE	*p*
Somatic comorbidity → fatigue	0.311	0.095	<0.001
Depressive symptoms → fatigue	0.726	0.042	<0.001
Accommodative coping → fatigue	0.117	0.054	0.030
Perceived health → fatigue	-0.157	0.056	0.004
**Indirect effects**	β	SE	*p*
Female gender → depression → fatigue	0.044	0.026	0.085
Education → perceived health → fatigue	-0.022	0.011	0.044
Comorbidity → perceived health → fatigue	0.080	0.032	0.012

β standardized path coefficient (StdYX for continuous variables, StdY for dichotomous variables), SE standard error.

Furthermore, education and somatic comorbidity had significant indirect effects on fatigue through mediation by perceived health. Having somatic comorbidity and lower education were significantly associated with lower perceived health, which in turn was associated with greater fatigue.

### Hypothesized model for participants with visual impairment

**Fig 2:**
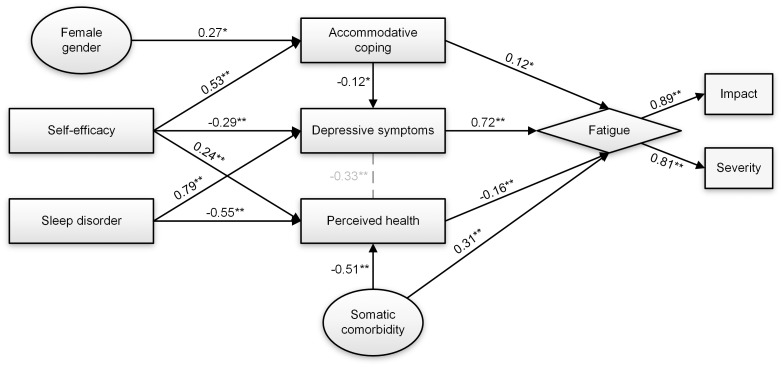
Path analysis output for the final model in the sample with visual impairment (*n* = 247). Direct effects are represented by arrows with standardized regression coefficients (StdYX for continuous variables, StdY for dichotomous variables. Grey dashed lines signify standardized residual covariance between variables. Ellipses represent independent variables. Rectangles represent psychosocial constructs. Constructs of the latent fatigue variable (diamond shape) are shown in dotted boxes. * *p*<0.05. ** *p*<0.01.

The final exploratory model was expanded with self-efficacy as an indirect variable because it was hypothesized to decrease depression and to increase accommodative coping and perceived health [32, 33]. Likewise, sleep disorder was included as an independent variable because it was expected to have a negative association with perceived health and a positive association with depression [34]. All new hypothesized pathways were confirmed, but Initial fit (data not shown) was not optimal. Therefore, further model refinements were made by elimination of education because of its non-significant pathway to perceived health. In addition, we allowed the residual covariance of depression and health status to correlate. Inspection of the fit indices suggested good fit for the final model (Table 4), explaining 64% of the total variance of fatigue. This model is graphically illustrated in Fig 2, standardized beta coefficients of the included direct and indirect pathways are summarized in Table 6. In addition to the direct effects of depression, accommodative coping, perceived health and somatic comorbidity, there were significant indirect associations between fatigue and the following factors: self-efficacy (total indirect effect: β = -0.228, *p*<0.001), sleep disorder (total indirect effect: β = 0.656, *p*<0.001) and accommodative coping (β = -0.089, *p*<0.037). The presence of sleep disorder was associated with higher levels of depressive symptoms and lower perceived health, which in turn were related to higher symptoms of fatigue. The positive indirect association between self-efficacy and fatigue was mediated by accommodative coping, more self-efficacy is related to more accommodative coping which is related to greater fatigue. In contrast, more self-efficacy is associated with less depressive symptoms and more positive perceived health, which is indirectly related to less fatigue. Moreover, greater tendencies of accommodative coping was indirectly associated with less fatigue via mediation by less symptoms of depression.

**Table 6:** Standardized direct and indirect path coefficients of the final hypothesized model for adults with visual impairment (n = 247).

**Direct effects**	β	SE	*p*
Somatic comorbidity → fatigue	0.311	0.095	0.001
Depressive symptoms → fatigue	0.723	0.042	<0.001
Accommodative coping → fatigue	0.116	0.053	0.030
Perceived health → fatigue	-0.158	0.055	0.004
**Indirect effects**	β	SE	*p*
Female gender → accommodative coping → fatigue	0.031	0.020	0.126
Comorbidity → perceived health → fatigue	0.081	0.032	0.011
Sleep disorder → depression → fatigue	0.569	0.109	<0.001
Sleep disorder → perceived health → fatigue	0.087	0.040	0.030
Self-efficacy → depression → fatigue	-0.205	0.047	<0.001
Self-efficacy → accommodative coping → fatigue	0.061	0.028	0.029
Self-efficacy → perceived health → fatigue	-0.037	0.015	0.015
Accommodative coping → depression → fatigue	-0.089	0.043	0.037

β standardized path coefficient (StdYX for continuous variables, StdY for dichotomous variables), SE standard error.

### Model examination in the reference group

**Fig 3:**
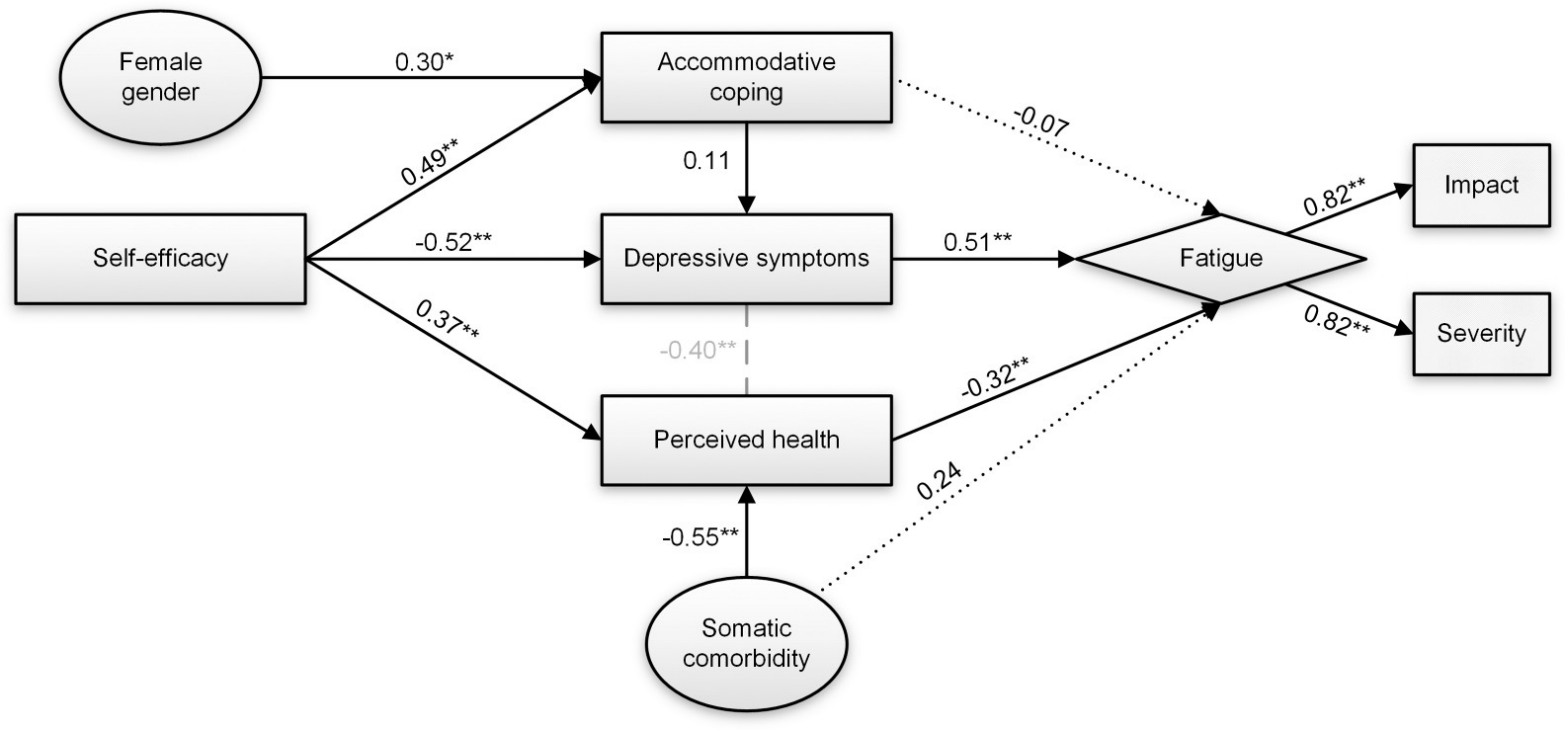
Path analysis output for the final model in the best fitting reference group subset (n = 151). Direct effects are represented by arrows with standardized regression coefficients (StdYX for continuous variables, StdY for dichotomous variables). Grey dashed lines signify standardized residual covariance between variables. Ellipses represent independent variables. Rectangles represent psychosocial constructs. Constructs of the latent fatigue variable (diamond shape) are shown in dotted boxes. * p<0.05. ** p<0.01.

In general, the final model demonstrated poor fit on all five generated normal-sight subsets (Table 4): SRMR indices were good, CFI values were borderline acceptable but TLI and RMSEA remained insufficient in all subsets. This was caused by the low number of persons (n = 2) in the reference group that reported sleep disorders. Exclusion of sleep disorder from the model resulted in good fit explaining 58% of the variance in fatigue (Table 4). Fig 3 shows that in the best performing subset, fatigue was directly related to depressive symptoms (β = 0.511, *p*<0.001) and perceived health (β = -0.322, *p*<0.001), but somatic comorbidity (β = 0.243, *p* = 0.085) and accommodative coping (β = -0.073, *p* = 0.341) did not reach significance. Similar to the target population, self-efficacy had a beneficial indirect effect (total indirect effect: β = -0.391, *p*<0.001) on fatigue mediated by depressive symptoms and perceived health, but not through accommodative coping. Moreover, the pathway from accommodative coping to depressive symptoms was insignificant.

## Discussion

In the present study, multidimensional path models were developed to identify the determinants of fatigue in adults with visual impairment by means of SEM. The results of our final model showed that a complex interplay between various psychological and health-related factors explained 64% of the variance in fatigue in adults with visual impairment. Overall, our novel findings suggest that depressive symptoms, and to a lesser extent perceived health, somatic comorbidity and accommodative coping contribute directly to fatigue. Self-efficacy was indirectly associated with lower fatigue by mediation of depressive symptoms and perceived health, whereas sleep disorder was indirectly associated with higher fatigue through its effects on depressive symptoms, accommodative coping and perceived health.

The second aim of our study was to compare the determinants of fatigue in adults with visual impairment to those of adults with normal sight. To this end, we applied the final model to data of the normally sighted reference group which initially resulted in poor fit. Model fit was subsequently improved by removal of sleep disorder because pathways with low variability often fail to make a positive impact on model performance. Only 1% of the reference group reported insomnia, hypersomnia or CRSD compared to 12% in participants with visual impairment. Inspection of the pathways and model coefficients in both populations highlighted some important similarities and differences with regard to fatigue associations. Depressive symptomatology and perceived health seem to directly contribute to fatigue both for individuals with visual impairment and normal sight. Also with regard to mediation of self-efficacy there were similar indirect effects in both populations. In contrast, the direct pathways of accommodative coping and sleep disorder, and the indirect pathways of sleep disorder were not found to be associated with fatigue in adults with normal sight. These factors could therefore potentially be specific to fatigue in patients with visual impairment.

Consistent with previous modeling studies in multiple sclerosis [35] and rheumatoid arthritis [4], our results show an important direct association between depressive symptomatology and fatigue in patients with visual impairment. Even though we excluded patients with a clinical diagnosis of depression, subclinical symptoms proved to be the strongest factor with the largest effect size in our models. This is not surprising because adults with visual impairment often experience mild but clinically relevant symptoms of depression, with an estimated prevalence rate of 32% compared to 12% in normally sighted peers [16]. While fatigue can be a clinical symptom of depression, its reciprocity with emotional distress has been described by patients, i.e. emotional dis-functioning and negative cognitions were reported to be both causes or consequences of fatigue in visual impairment [36].

In our study we found that sleep disorders were not directly related to fatigue in adults with visual impairment, but had a substantial indirect pathway mediated by depressive symptomatology. This finding is consistent with previous studies in cancer patients and indicates that sleep disorders may increase fatigue by exacerbating depressive symptoms [37, 38]. Sleep disturbances are traditionally conceptualized as secondary manifestations of mood disorders, but recent evidence from longitudinal studies strongly suggest that sleep disturbances may be independent risk factors for depression [39]. For example, the presence of insomnia during adolescence has been reported to predict depression in young adulthood and has been argued to increase the severity of depression [40]. Difficulties with emotion regulation induced by sleep disturbances have been described to play a critical role in this relationship [41]. This could explain the observed mediation in our study because people with visual impairment often experience emotional distress in response to the challenges induced by vision loss [42]. In turn, depressed mood can cause fatigue, as has been reported by people with visual impairment, due to feelings of inferiority, unrealistic expectations and negative thoughts about vision loss and related disability [36].

In contrast to the established literature in chronic disease, we found a positive relationship between accommodative coping and fatigue in the sample with visual impairment. Individuals with higher tendencies of accommodative coping experienced more fatigue in our study, whereas previous studies have described positive aspects of accommodative coping. For example, the ability to flexibly adjust personal goals to situational constraints and challenges has been stressed as an important resource for well-being in old age [43]. Also, with regard to coping with visual disability the importance of flexible goal adjustment has been stressed for improving mental health and depressive symptoms [44]. The positive association found in our study could be interpreted as coping efforts in response to substantial fatigue. In this sense, higher tendencies of flexible adjustment reflect severe levels of fatigue and may therefore not suffice as a coping strategy. This notion is supported by data from a qualitative perspective, where several coping strategies described by adults with visual impairment appeared to be unsuccessful in handling fatigue [36]. On the other hand, our relatively large sample size may have caused some unexpected associations in our models and the standardized direct effect of accommodative coping was relatively low compared to other direct pathways in the model. It should also be noted that flexible coping had a negative pathway to fatigue via depression, indicating a beneficial role of coping by reduction of depression. Nevertheless, more studies are needed to shed light on our conflicting results between accommodative coping and fatigue.

The final hypothesized model also identified self-efficacy as an important construct associated with fatigue that was mediated by several psychological factors. More self-efficacy was related to more accommodative coping, greater perceived health and less depressive symptoms, which in turn were associated with lower fatigue levels. These findings suggest that self-efficacy may be an important modifiable target for interventions to improve coping effects for dealing with fatigue. This notion extents to previous intervention studies that have demonstrated the importance of self-efficacy with regard to coping and fatigue improvements in chronic disease [45, 46].

Nevertheless, there may be other factors relevant in explaining vision-related fatigue that were not included in the present study. For example, several other constructs such as illness perceptions, catastrophizing thoughts, anxiety and disease-related activity have been related to increased fatigue in previous analyses. Furthermore, modifiable life style factors such as alcohol use and dietary habits have been stressed as predictors of fatigue in multiple sclerosis [47]. Future studies are therefore needed to explore these factors. Regarding vision-specific factors, the influence of light, visual perception and adaptation to vision loss which have been reported by patients could also potentially explain fatigue in people with visual impairment [36].

A strength of our study was the inclusion of IRT models to ensure psychometric properties of our outcome measures. Most of the questionnaires provided adequate fit to the IRT models after merging response categories and/or removal of poor fitting items. Furthermore, the inclusion of a reference group allowed for a comparison between the fatigue determinants in case of vision loss and normal sight. However, our snowball sampling method may have been suboptimal because it resulted in a young subpopulation within the reference group. Alternative methods such as matching reference in terms of age and gender could have been more effective to prevent these kinds of sampling issues. In our study we accounted for the bimodal age distribution in the reference group by selecting 10 participants from the younger subset. This random selection was repeated five times to account for bias in estimations. Although this method may be considered suboptimal we believe it was necessary to allow examination of our model in the reference group. Another limitation of our study was that for some questionnaires (i.e. G-SES-12, USER-P, GAS-Re, GAS-De) it was not possible to fit an IRT model. In these cases, regular summary scores were used as potential explanatory variables or mediators. The psychometric properties of these scales may have been unsatisfactory and could have influenced the results of our analyses. Furthermore, due to skewness of the HSDQ subscale data, it was not possible to examine the relation between fatigue and severity of the individual sleep disorders. However, the dichotomization of the subscales enabled us to evaluate the presence of one or more sleep disorders in the fatigue models for participants with visual impairment. Finally, we are aware of the criticism raised by some researchers in psychology and social sciences on testing mediation when based on cross-sectional data [48, 49]. The lack of time precedence has been proposed as one of the most important limitations. When all variables are measured simultaneously, it is impossible to determine a temporal order of the assumed causes, mediators and outcomes. It is therefore argued that cross-sectional designs are not appropriate to establish causal interference because directionality is solely based on the researcher’s assumptions [48, 49]. Nevertheless, mediation models on cross-sectional data are common and it was possible to disentangle some (in)direct associations between various relevant factors and fatigue in a population with visual impairment. Moreover, the pathways identified by our study are supported by theoretical frameworks from research in chronic patient populations and might have generated hypotheses for future studies [11, 32–34]. Longitudinal studies are recommended to investigate the suggested causal directions in future and to investigate which factors predict vision-related fatigue over time.

### Clinical implications

The multidimensional fatigue model developed in the present study showed that fatigue in adults with visual impairment was associated with multiple generic and specific factors. Knowledge of these factors may aid future studies in developing treatment options for vision-related fatigue. Our findings could be relevant for clinical practice, suggesting that depression and mood disturbances can be addressed to improve fatigue and related disability in adults with visual impairment. Psychological interventions such as cognitive behavioral therapy (CBT) are effective treatment options for depression [50], although the evidence is less convincing in the field of low vision [51]. Treatment of insomnia, hypersomnia and CRSD also warrants special attention considering its specific contribution to fatigue in visual impairment, and may improve fatigue by having a positive effect on mood [52]. A recent meta-analysis indicates that comorbid sleep disorders in patients with medical or psychiatric disorders can be effectively treated with CBT [53]. However the efficacy of these interventions have not been evaluated in persons with vision loss and should be addressed in future studies. Alternatively, self-management programs may especially be suitable considering the important role of self-efficacy in coping and fatigue. Internet-based E-Health interventions could also be considered because they can be flexibly adapted to the requirements of individual patients [54]. Healthcare providers should be aware of the direct association between somatic comorbidity and fatigue, as this may reinforce experienced disability and could hinder the rehabilitation process. For rehabilitation services, a multidisciplinary holistic approach is advised to account for the multifactorial nature of fatigue. Finally, the results of our model may facilitate communication about fatigue between patient and health care provider.

## Supporting information

S1 Table Standardized direct path coefficients of the first exploratory model for adults with visual impairment (*n* = 247). β standardized path coefficient (StdYX for continuous variables, StdY for dichotomous variables), SE standard error.
(DOCX)
