# Supplementary material for: Understanding fatigue in adults with visual impairment: A path analysis study of sociodemographic, psychological and health-related factors
Source: PLoS One. 2019 Oct 25;14(10):e0224340. doi: 10.1371/journal.pone.0224340 (PMC6814229; doi:10.1371/journal.pone.0224340)
Supplement: S1 Table — β standardized path coefficient (StdYX for continuous variables, StdY for dichotomous variables), SE standard error. (DOCX) [file pone.0224340.s001.docx]

**S1 Table.**

| **Direct effects** | β | SE | *p* |
| --- | --- | --- | --- |
|  |  |  |  |
| Potential mediating variables |  |  |  |
| Depressive symptoms → fatigue | 0.720 | 0.047 | **<0.001** |
| Self-efficacy → fatigue | -0.109 | 0.082 | 0.182 |
| Frequency of participation → fatigue | -0.027 | 0.056 | 0.636 |
| Goal re-engagement → fatigue | -0.039 | 0.064 | 0.543 |
| Goal dis-engagement → fatigue | 0.058 | 0.054 | 0.284 |
| Accommodative coping → fatigue | 0.165 | 0.070 | **0.018** |
| Assimilative coping → fatigue | 0.013 | 0.074 | 0.864 |
| Perceived health status → fatigue | -0.155 | 0.060 | **0.009** |
| Sleep disorder → fatigue | 0.146 | 0.148 | 0.323 |
|  |  |  |  |
| Independent variables |  |  |  |
| Gender → fatigue | 0.064 | 0.097 | 0.511 |
| Age → fatigue | -0.007 | 0.057 | 0.901 |
| Education → fatigue | 0.039 | 0.053 | 0.464 |
| Living situation → fatigue | 0.042 | 0.101 | 0.692 |
| Somatic comorbidity → fatigue | 0.324 | 0.101 | **0.001** |
| Working status → fatigue | -0.070 | 0.129 | 0.591 |
|  |  |  |  |
